# 4,4-Dimethyl-1-(3-nitro­phen­yl)pent-1-en-3-one

**DOI:** 10.1107/S1600536809018479

**Published:** 2009-05-20

**Authors:** Ping Chen, Lin Xia, Ai-Xi Hu, Jiao Ye

**Affiliations:** a College of Chemical and Biological Engineering, Changsha University of Science and Technology, 410004 Changsha, People’s Republic of China; b College of Chemistry and Chemical Engineering, Hunan University, 410082 Changsha, People’s Republic of China

## Abstract

All the non-hydrogen atoms except one methyl C atom of the title compound, C_13_H_15_NO_3_,  lie on a crystallographic mirror plane perpendicular to the *b* axis. The crystal packing is stabilized by two weak inter­molecular C—H⋯O hydrogen bonds.

## Related literature

The title compound is an important intermediate in the pesticides industry (Wang *et al.*, 2006 ▶). For related structures, see: Anuradha *et al.* (2008 ▶); Butcher *et al.* (2007 ▶); Gong *et al.* (2008 ▶); Harrison *et al.* (2007 ▶); Patil *et al.* (2007 ▶); Sarojini *et al.* (2007 ▶); Thiruvalluvar *et al.* (2007 ▶, 2008 ▶); Xia & Hu (2008 ▶).
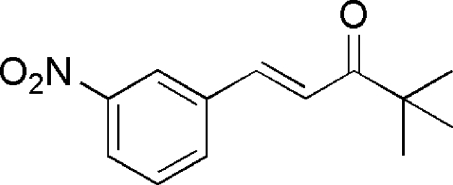


## Experimental

### 

#### Crystal data

C_13_H_15_NO_3_
*M* *_r_* = 233.26
Orthorhombic, 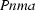

*a* = 11.3375 (9) Å
*b* = 7.2163 (6) Å
*c* = 14.9327 (12) Å
*V* = 1221.72 (17) Å^3^
*Z* = 4
Mo *K*α radiation
μ = 0.09 mm^−1^
*T* = 173 K
0.48 × 0.36 × 0.15 mm

#### Data collection

Bruker SMART 1000 CCD diffractometer
Absorption correction: multi-scan (*SADABS*; Sheldrick, 2004 ▶) *T*_min_ = 0.958, *T*_max_ = 0.987
5485 measured reflections
1280 independent reflections
937 reflections with *I* > 2σ(*I*)
*R*_int_ = 0.030

#### Refinement

*R*[*F* ^2^ > 2σ(*F* ^2^)] = 0.041
*wR*(*F* ^2^) = 0.123
*S* = 1.06
1280 reflections
101 parameters
H-atom parameters constrained
Δρ_max_ = 0.26 e Å^−3^
Δρ_min_ = −0.16 e Å^−3^

### 

Data collection: *SMART* (Bruker, 2001 ▶); cell refinement: *SAINT-Plus* (Bruker, 2003 ▶); data reduction: *SAINT-Plus*; program(s) used to solve structure: *SHELXS97* (Sheldrick, 2008 ▶); program(s) used to refine structure: *SHELXL97* (Sheldrick, 2008 ▶); molecular graphics: *SHELXTL* (Sheldrick, 2008 ▶); software used to prepare material for publication: *SHELXTL*.

## Supplementary Material

Crystal structure: contains datablocks I, global. DOI: 10.1107/S1600536809018479/bt2958sup1.cif

Structure factors: contains datablocks I. DOI: 10.1107/S1600536809018479/bt2958Isup2.hkl

Additional supplementary materials:  crystallographic information; 3D view; checkCIF report

## Figures and Tables

**Table 1:** Hydrogen-bond geometry (Å, °)

*D*—H⋯*A*	*D*—H	H⋯*A*	*D*⋯*A*	*D*—H⋯*A*
C6—H6*A*⋯O2^i^	0.98	2.44	3.367 (2)	158
C10—H10⋯O2^ii^	0.95	2.50	3.366 (3)	152

Symmetry codes: (i) 
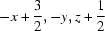
; (ii) 
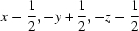
.

**Fig. 1.**
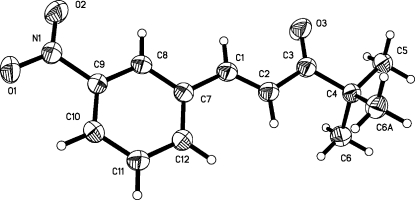
Molecular structure of the title compound, showing 30% probability displacement ellipsoids.

**Fig. 2.**
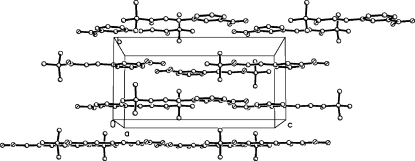
Packing diagram of the title compound.

## supplementary crystallographic information

### Comment

The title compound, 1-(3-nitrophenyl)-4,4-dimethylpentan-3-one, is a very important intermediate in the pesticides industry (Wang *et al.*, 2006). Continuing our work (Xia & Hu, 2008), we report the synthesis and crystal structure of the title compound. Several crystal structures containing phenylprop-2-en-1-one moiety have been recently published (Anuradha *et al.*, 2008; Butcher *et al.*, 2007; Gong *et al.*, 2008; Harrison *et al.*, 2007; Patil *et al.*, 2007; Sarojini *et al.*, 2007; Thiruvalluvar *et al.*, 2007, 2008; Xia & Hu, 2008). In the title compound (Fig. 1), the carbonyl, ethenyl and nitro groups are coplanar with the benzene ring. The bond lengths and bond angles in (I) are in excellent agreement with the corresponding bond lengths and angles reported in the related compounds given above. The crystal packing is illustrated in Fig. 2.

### Experimental

3,3-dimethylbutan-2-one(0.0105 mol) was added dropwise into the solution of 3-nitrobenzaldehyde (0.01 mol) and 60 ml ethanol. Then  0.1 g 50% NaOH solution as catalyzer was added and the solution was stirred at 333 K for 5 h (monitored by TLC). Part of the solvent was evaporated and the solution was cooled to 277 K and a precipitate formed. It was filtered and dried. Crystals suitable for X-ray structure determination were obtained by slow evaporation of an ethanol solution at room temperature.

### Refinement

H atoms were positioned geometrically (C—H = 0.95 or C_methyl_-H  = 0.98 Å) with U_iso_(H) = 1.2U_eq_(C) or 1.5U_eq_(C_methyl_). The methyl group on a general position was allowed to rotate but not to tip.

### Crystal data

**Table:**

C_13_H_15_NO_3_	*D*_x_ = 1.268 Mg m^−^^3^
*M**_r_* = 233.26	Melting point: 363 K
Orthorhombic, *P**n**m**a*	Mo *K*α radiation, λ = 0.71073 Å
Hall symbol: -P 2ac 2n	Cell parameters from 1920 reflections
*a* = 11.3375 (9) Å	θ = 2.3–27.7°
*b* = 7.2163 (6) Å	µ = 0.09 mm^−^^1^
*c* = 14.9327 (12) Å	*T* = 173 K
*V* = 1221.72 (17) Å^3^	Block, colourless
*Z* = 4	0.48 × 0.36 × 0.15 mm
*F*(000) = 496	

### Data collection

**Table:**

Bruker SMART 1000 CCD diffractometer	1280 independent reflections
Radiation source: fine-focus sealed tube	937 reflections with *I* > 2σ(*I*)
graphite	*R*_int_ = 0.030
ω scans	θ_max_ = 26.0°, θ_min_ = 2.3°
Absorption correction: multi-scan (*SADABS*; Sheldrick, 2004)	*h* = −13→12
*T*_min_ = 0.958, *T*_max_ = 0.987	*k* = −3→8
5485 measured reflections	*l* = −18→17

### Refinement

**Table:**

Refinement on *F*^2^	Primary atom site location: structure-invariant direct methods
Least-squares matrix: full	Secondary atom site location: difference Fourier map
*R*[*F*^2^ > 2σ(*F*^2^)] = 0.041	Hydrogen site location: inferred from neighbouring sites
*wR*(*F*^2^) = 0.123	H-atom parameters constrained
*S* = 1.06	*w* = 1/[σ^2^(*F*_o_^2^) + (0.0591*P*)^2^ + 0.3634*P*] where *P* = (*F*_o_^2^ + 2*F*_c_^2^)/3
1280 reflections	(Δ/σ)_max_ = 0.001
101 parameters	Δρ_max_ = 0.26 e Å^−^^3^
0 restraints	Δρ_min_ = −0.16 e Å^−^^3^

### Special details

**Table:**

Geometry. All e.s.d.'s (except the e.s.d. in the dihedral angle between two l.s. planes) are estimated using the full covariance matrix. The cell e.s.d.'s are taken into account individually in the estimation of e.s.d.'s in distances, angles and torsion angles; correlations between e.s.d.'s in cell parameters are only used when they are defined by crystal symmetry. An approximate (isotropic) treatment of cell e.s.d.'s is used for estimating e.s.d.'s involving l.s. planes.
Refinement. Refinement of *F*^2^ against ALL reflections. The weighted *R*-factor *wR* and goodness of fit *S* are based on *F*^2^, conventional *R*-factors *R* are based on *F*, with *F* set to zero for negative *F*^2^. The threshold expression of *F*^2^ > σ(*F*^2^) is used only for calculating *R*-factors(gt) *etc*. and is not relevant to the choice of reflections for refinement. *R*-factors based on *F*^2^ are statistically about twice as large as those based on *F*, and *R*- factors based on ALL data will be even larger.

### Fractional atomic coordinates and isotropic or equivalent isotropic displacement parameters (Å^2^)

**Table:**

	*x*	*y*	*z*	*U*_iso_*/*U*_eq_	Occ. (<1)
C1	0.64133 (19)	0.2500	0.12364 (15)	0.0363 (6)	
H1	0.7249	0.2500	0.1281	0.044*	
C2	0.5825 (2)	0.2500	0.19956 (15)	0.0371 (6)	
H2	0.4987	0.2500	0.1983	0.045*	
C3	0.6446 (2)	0.2500	0.28774 (15)	0.0367 (6)	
C4	0.56797 (18)	0.2500	0.37208 (15)	0.0317 (5)	
C5	0.6474 (2)	0.2500	0.45437 (16)	0.0392 (6)	
H5A	0.5987	0.2500	0.5086	0.059*	
H5B	0.6973	0.3609	0.4537	0.059*	0.50
H5C	0.6973	0.1391	0.4537	0.059*	0.50
C6	0.48919 (15)	0.0777 (3)	0.37212 (12)	0.0419 (5)	
H6A	0.5385	−0.0338	0.3701	0.063*	
H6B	0.4374	0.0803	0.3196	0.063*	
H6C	0.4412	0.0761	0.4267	0.063*	
C7	0.59173 (19)	0.2500	0.03248 (15)	0.0310 (5)	
C8	0.66910 (19)	0.2500	−0.03967 (15)	0.0313 (5)	
H8	0.7519	0.2500	−0.0299	0.038*	
C9	0.62418 (18)	0.2500	−0.12592 (14)	0.0296 (5)	
C10	0.5051 (2)	0.2500	−0.14355 (15)	0.0348 (6)	
H10	0.4764	0.2500	−0.2033	0.042*	
C11	0.42813 (19)	0.2500	−0.07129 (16)	0.0381 (6)	
H11	0.3454	0.2500	−0.0814	0.046*	
C12	0.47098 (19)	0.2500	0.01538 (15)	0.0363 (6)	
H12	0.4171	0.2500	0.0641	0.044*	
N1	0.70773 (17)	0.2500	−0.20150 (12)	0.0359 (5)	
O1	0.66864 (16)	0.2500	−0.27777 (11)	0.0501 (5)	
O2	0.81309 (15)	0.2500	−0.18457 (12)	0.0570 (6)	
O3	0.75145 (14)	0.2500	0.29171 (11)	0.0559 (6)	

### Atomic displacement parameters (Å^2^)

**Table:**

	*U*^11^	*U*^22^	*U*^33^	*U*^12^	*U*^13^	*U*^23^
C1	0.0277 (11)	0.0469 (15)	0.0344 (13)	0.000	−0.0032 (9)	0.000
C2	0.0283 (11)	0.0495 (15)	0.0337 (13)	0.000	−0.0012 (9)	0.000
C3	0.0319 (12)	0.0453 (15)	0.0328 (13)	0.000	−0.0006 (9)	0.000
C4	0.0315 (11)	0.0347 (13)	0.0290 (12)	0.000	−0.0002 (9)	0.000
C5	0.0373 (12)	0.0471 (15)	0.0333 (13)	0.000	−0.0019 (10)	0.000
C6	0.0415 (9)	0.0424 (10)	0.0418 (10)	−0.0048 (8)	0.0023 (7)	0.0001 (8)
C7	0.0304 (11)	0.0317 (13)	0.0310 (12)	0.000	−0.0015 (9)	0.000
C8	0.0265 (10)	0.0332 (13)	0.0340 (13)	0.000	−0.0030 (9)	0.000
C9	0.0312 (11)	0.0281 (12)	0.0297 (12)	0.000	0.0014 (9)	0.000
C10	0.0352 (12)	0.0387 (14)	0.0306 (12)	0.000	−0.0043 (9)	0.000
C11	0.0279 (11)	0.0494 (15)	0.0372 (13)	0.000	−0.0014 (9)	0.000
C12	0.0321 (12)	0.0447 (14)	0.0322 (13)	0.000	0.0006 (9)	0.000
N1	0.0355 (11)	0.0405 (12)	0.0316 (12)	0.000	0.0020 (8)	0.000
O1	0.0501 (10)	0.0725 (14)	0.0278 (10)	0.000	−0.0010 (8)	0.000
O2	0.0322 (9)	0.0955 (16)	0.0434 (11)	0.000	0.0058 (8)	0.000
O3	0.0300 (10)	0.1027 (17)	0.0350 (10)	0.000	−0.0011 (7)	0.000

### Geometric parameters (Å, °)

**Table:**

C1—C2	1.315 (3)	C6—H6C	0.9800
C1—C7	1.473 (3)	C7—C8	1.389 (3)
C1—H1	0.9500	C7—C12	1.393 (3)
C2—C3	1.493 (3)	C8—C9	1.385 (3)
C2—H2	0.9500	C8—H8	0.9500
C3—O3	1.213 (3)	C9—C10	1.376 (3)
C3—C4	1.530 (3)	C9—N1	1.474 (3)
C4—C5	1.523 (3)	C10—C11	1.388 (3)
C4—C6	1.531 (2)	C10—H10	0.9500
C4—C6^i^	1.531 (2)	C11—C12	1.382 (3)
C5—H5A	0.9800	C11—H11	0.9500
C5—H5B	0.9800	C12—H12	0.9500
C5—H5C	0.9800	N1—O2	1.221 (2)
C6—H6A	0.9800	N1—O1	1.222 (2)
C6—H6B	0.9800		
			
C2—C1—C7	127.1 (2)	C4—C6—H6C	109.5
C2—C1—H1	116.5	H6A—C6—H6C	109.5
C7—C1—H1	116.5	H6B—C6—H6C	109.5
C1—C2—C3	121.4 (2)	C8—C7—C12	118.6 (2)
C1—C2—H2	119.3	C8—C7—C1	118.40 (19)
C3—C2—H2	119.3	C12—C7—C1	123.0 (2)
O3—C3—C2	120.9 (2)	C9—C8—C7	119.27 (19)
O3—C3—C4	121.8 (2)	C9—C8—H8	120.4
C2—C3—C4	117.30 (19)	C7—C8—H8	120.4
C5—C4—C3	109.19 (18)	C10—C9—C8	122.6 (2)
C5—C4—C6	110.15 (12)	C10—C9—N1	118.97 (19)
C3—C4—C6	109.36 (12)	C8—C9—N1	118.42 (18)
C5—C4—C6^i^	110.15 (12)	C9—C10—C11	117.9 (2)
C3—C4—C6^i^	109.36 (12)	C9—C10—H10	121.0
C6—C4—C6^i^	108.63 (18)	C11—C10—H10	121.0
C4—C5—H5A	109.5	C12—C11—C10	120.5 (2)
C4—C5—H5B	109.5	C12—C11—H11	119.8
H5A—C5—H5B	109.5	C10—C11—H11	119.8
C4—C5—H5C	109.5	C11—C12—C7	121.1 (2)
H5A—C5—H5C	109.5	C11—C12—H12	119.4
H5B—C5—H5C	109.5	C7—C12—H12	119.4
C4—C6—H6A	109.5	O2—N1—O1	123.22 (19)
C4—C6—H6B	109.5	O2—N1—C9	118.06 (18)
H6A—C6—H6B	109.5	O1—N1—C9	118.73 (18)
			
C7—C1—C2—C3	180.0	C7—C8—C9—C10	0.0
C1—C2—C3—O3	0.0	C7—C8—C9—N1	180.0
C1—C2—C3—C4	180.0	C8—C9—C10—C11	0.0
O3—C3—C4—C5	0.0	N1—C9—C10—C11	180.0
C2—C3—C4—C5	180.0	C9—C10—C11—C12	0.0
O3—C3—C4—C6	−120.58 (12)	C10—C11—C12—C7	0.0
C2—C3—C4—C6	59.42 (12)	C8—C7—C12—C11	0.0
O3—C3—C4—C6^i^	120.58 (12)	C1—C7—C12—C11	180.0
C2—C3—C4—C6^i^	−59.42 (12)	C10—C9—N1—O2	180.0
C2—C1—C7—C8	180.0	C8—C9—N1—O2	0.0
C2—C1—C7—C12	0.0	C10—C9—N1—O1	0.0
C12—C7—C8—C9	0.0	C8—C9—N1—O1	180.0
C1—C7—C8—C9	180.0		

Symmetry codes: (i) *x*, −*y*+1/2, *z*.

### Hydrogen-bond geometry (Å, °)

**Table:**

*D*—H···*A*	*D*—H	H···*A*	*D*···*A*	*D*—H···*A*
C6—H6A···O2^ii^	0.98	2.44	3.367 (2)	158
C10—H10···O2^iii^	0.95	2.50	3.366 (3)	152

Symmetry codes: (ii) −*x*+3/2, −*y*, *z*+1/2; (iii) *x*−1/2, −*y*+1/2, −*z*−1/2.
